# Effect of Temperature on the Corrosion Behavior and Corrosion Resistance of Copper–Aluminum Laminated Composite Plate

**DOI:** 10.3390/ma15041621

**Published:** 2022-02-21

**Authors:** Qiannan Li, Yifan Zhang, Yulin Cheng, Xiaojiao Zuo, Yinxiao Wang, Xiaoguang Yuan, Hongjun Huang

**Affiliations:** School of Materials Science and Engineering, Shenyang University of Technology, Shenyang 110870, China; lz_liqiannan@163.com (Q.L.); zyf_sindy@163.com (Y.Z.); chengyulintc@163.com (Y.C.); yuanxg@sut.edu.cn (X.Y.); huanghong1977@163.com (H.H.)

**Keywords:** copper–aluminum laminated composite plate, neutral salt-spray corrosion, electrochemical corrosion, corrosion behavior, corrosion resistance, ionic conductivity

## Abstract

In this paper, the effect of temperature on the corrosion behavior and corrosion resistance of the copper–aluminum laminated composite plates were investigated by salt-spray corrosion, potential polarization curve and electrochemical impedance spectroscopy. Moreover, the microstructure of the copper–aluminum laminated composite plate after salt-spray corrosion was observed by scanning electron microscope, and X-ray photoelectron spectroscopy was used to study the composition of corrosion product. The results revealed that the corrosion products of the copper–aluminum laminated composite plate were Al_2_O_3_ and AlOOH. Due to the galvanic corrosion of the copper–aluminum laminated composite plate, the cathode underwent oxygen absorption corrosion during the corrosion process; therefore, the presence of moisture and the amount of dissolved oxygen in the corrosive environment had a great influence on the corrosion process. The increasing temperature would evaporate a large amount of moisture, resulting in the corrosion product—aluminum oxide dehydrated and covered the surface of the material in the process of salt-spray corrosion, which played a role in protecting the material. Therefore, the corrosion resistance of the copper–aluminum laminated composite plate first decreased and then increased. In the salt-spray corrosion environment, the corrosion resistance of the copper–aluminum laminated composite plate reached the lowest at 45 °C, and its corrosion rate was the fastest, at 0.728 g/m^2^·h. The electrochemical corrosion occurred in the solution, and the impact was small; however, in addition to the protective corrosion products, the ion mobility in the solution also had a certain influence on the corrosion rate, and the ionic activity increased with the increase of temperature. Therefore, the corrosion resistance of the copper–aluminum laminated composite plate gradually decreased as the temperature increased, and its corrosion resistance was the worst at 50 °C.

## 1. Introduction

Copper–aluminum laminated composite plates were widely applied to the chemical industry and electronics, due to their exceptional advantages, including superior electrical and thermal conductivity, favorable processing performance, and cut-price [1,2,3,4]. As an extraordinary potential material, the commercialization of it would substitute for pure copper, thereby reducing the use and consumption of copper resources [5,6]. However, the existence of the standard electrode potential difference between copper and aluminum—that aluminum had a low standard electrode potential (−1.662 V) compared with copper (+0.342 V)—made the copper–aluminum laminated composite plate experience severe galvanic corrosion easily when it was in a humid or corrosive medium environment. Aluminum underwent an oxidation reaction as an anode and corroded rapidly, causing a poor corrosion resistance and lessening its service life [7,8], which probably caused engineering failure or safety hazards [9,10].

It was found that pitting corrosion appeared on the aluminum matrix first when the copper–aluminum galvanic couple was brought into contact with the corrosive medium [11,12,13]. As in the marine environment with high Cl^−^ concentration, severe pitting corrosion and peeling would initiate from the aluminum in the copper–aluminum galvanic couple, and it would develop into crevice corrosion along the interface between aluminum and copper [14,15]. Within the specified corrosion time, the entire surface of the aluminum matrix eventually was corroded, starting from the interface and extending to the depths [16]. It was demonstrated that multiple pitting pits appeared on the surface of the material within a short period of time in an aerobic environment; the corrosion reaction occurred rapidly and violently, yet basically the galvanic corrosion would not happen in materials without oxygen, and the corrosion potential did not fluctuate [11,17]. Pu et al. [18] investigated the fretting-corrosion behavior of 6082 aluminum alloy in 3.5% NaCl solution; they found that the wear was more serious in places with high oxygen content and that the corrosion would be weakened due to lack of oxygen. Apparently, although the copper–aluminum laminated composite material underwent galvanic corrosion, its corrosion behavior changed with the different service environment.

As a conductor, copper–aluminum busbar was used for the transmission circuit frequently. Due to Joule’s law, Q = I^2^Rt, the temperature would increase when the current was passing through. It was proved that the surface temperature of the copper–aluminum increased from 31 to 49 °C with the increasing current from 180 to 300 A [19]. With the temperature increased, the diffusion rate increased and synchronously the electrolyte resistance decreased, accelerating the corrosion. It was illustrated that the corrosion weight loss or corrosion rate was increased significantly with the increasing temperature [20,21]. Nonetheless, sometimes if the temperature exceeded a certain limit, the corrosion rate would reduce as well [22]. However, the influence of temperature on the corrosion behavior and corrosion mechanism of the galvanic corrosion of copper–aluminum laminated composite plates was still unknown.

Therefore, this work focused on the corrosion behavior and corrosion mechanism of copper–aluminum laminated composite plates in a salt-spray environment and electrochemical corrosion environment at 30, 35, 40, 45, and 50 °C. The microscopic morphology, corrosion rate, electrochemical corrosion resistance, ion mobility, and corrosion pit depth of the copper–aluminum laminated composite plate after corrosion were delved. The study aimed to clarify the effect of temperature on the corrosion behavior of copper–aluminum laminated composite plates in order to evaluate the performance and service life of the copper–aluminum laminated composite plate, and it provided scientific basis to solve or improve the corrosion issues of copper–aluminum laminated composite plate in application.

## 2. Materials and Methods

### 2.1. Sample

This experiment used a copper–aluminum laminated composite plate made of 1050 industrial pure aluminum and industrial pure copper T2 cast and rolled that was a sandwich plate with the copper width of 1 mm on both sides and the aluminum width of 8 mm in the middle. The copper–aluminum-copper laminated composite plate was processed into a sample with the size of 20 × 10 × 20 mm^3^, polished, and then weighted. The processed sample should be used immediately in order to prevent contamination in the environment.

### 2.2. Salt-Spray Corrosion Test

The sample was exposed to the 5% NaCl salt-spray solution for 24 h at 30, 35, 40, 45, and 50 °C. We kept the inclination angle of the sample suspended at 20° and the distance between the working surface to the nozzle same in the salt-spray corrosion chamber (SF-MIT90C, Changzhou Sanfeng, Changzhou, China). The three samples after salt-spray corrosion were cleaned according to GB/T 16545-2015; we then measured the weight loss and took the average value. The corrosion morphology of sample with corrosion products was observed by using scanning electron microscope (SEM, Hitachi S-3400N, Japan Rili, Tokyo, Japan).

The X-ray photoelectron spectrometer (XPS, Thermo SCIENTIFIC ESCALAB 250Xi, Thermo Fisher Scientific, Waltham, MA, USA) was used to study the corrosion product phases formed on the surface of the copper–aluminum laminated composite plate after corrosion, and the XPS spectra were analyzed by using Thermo Avantage (v5.9922, 2020, Thermo Fisher Scientific, Waltham, MA, USA) and Casa XPS software (2.3.23, 2020, Caltech, Pasadena, CA, USA).

### 2.3. Electrochemical Test

The electrochemical workstation (CHI604E, Shanghai Chenhua, Shanghai, China) was used to measure the open circuit potential (OCP), electrochemical impedance spectroscopy (AC impedance), and potential polarization curve (Tafel) of the sample. The electrochemical test used a three-electrode system, in which the working electrode was a cross-section with 1 × 1 cm^2^ of the copper–aluminum laminated composite plate, the auxiliary electrode was a platinum wire, and the reference electrode was saturated calomel. The electrolytic cell with 3.5% NaCl solution was respectively placed in a constant-temperature water-bath vessel (BHS-1, Shanghai Lingke) at 30, 35, 40, 45, and 50 °C. The open circuit potential test time was 1800 s, which was long enough to ensure that the sample was in a stable state in the electrolyte. The frequency range of AC impedance test was from 10^5^ to 10^−2^ Hz, and the sine wave disturbance amplitude was 10 mV/s. The potential scanning range of the polarization curve was −1 to +1 V versus OCP, and the sweep rate was 0.1667 mV/s. The sensitivity of the tests was set to automatically adjust to prevent overload.

In order to explore the influence of the electrons mobility on the electrochemical corrosion rate of the copper–aluminum laminated composite plate, the beaker containing the 3.5% NaCl solution was put into the constant-temperature water-bath vessel at 30, 35, 40, 45, and 50 °C, respectively, using a high-precision conductivity meter (AZ8362, Taiwan Hengxin) to measure the change in the conductivity of the electrolyte at different temperatures within 24 h.

## 3. Results

### 3.1. Microstructure Analysis

**Figure 1:**
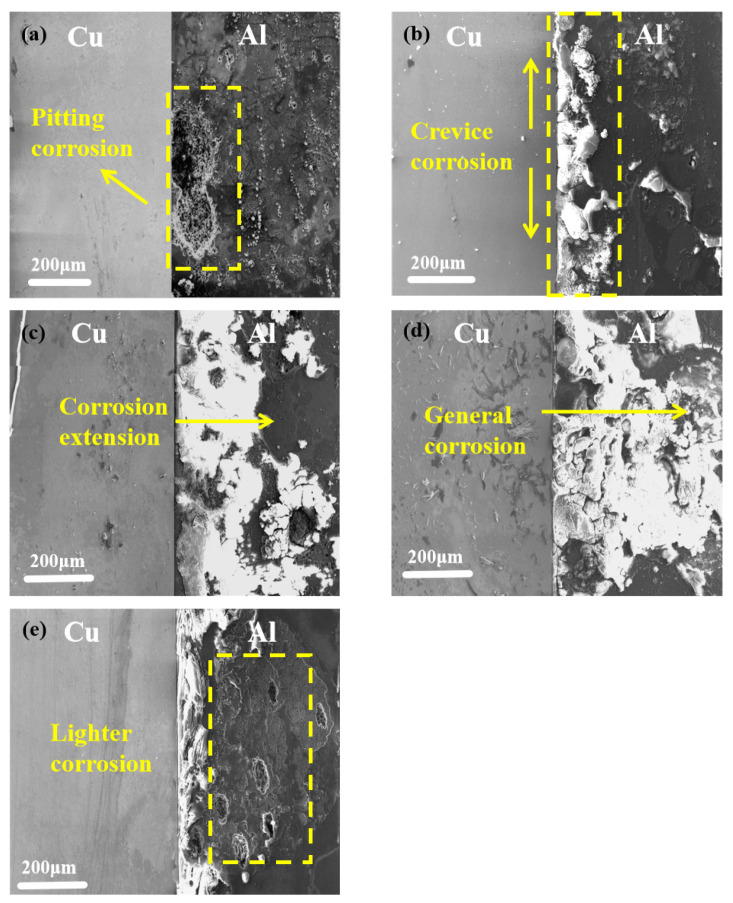
Microscopic morphology of the copper–aluminum laminated composite plate after corrosion for 24 h in a salt-spray environment: (**a**) 30, (**b**) 35, (**c**) 40, (**d**) 45, and (**e**) 50 °C.

Figure 1 shows the morphology of the cross-section of the copper–aluminum laminated composite plate after salt-spray corrosion at different temperatures for 24 h. Figure 1a shows the corrosion initiated from the aluminum side of the interface of the copper–aluminum laminated composite plate at 30 °C in the form of pits that are 200 microns in length. When the temperature was increased to 35 °C and above, the pitting corrosion increased and expanded. First, cracks appeared at the copper–aluminum interface to form crevice corrosion and then further expanded to the center of the aluminum matrix to the entire cross-section, and finally general corrosion formed. However, with the temperature further rising up to 50 °C, as shown in Figure 1e, crevice corrosion appeared accompanied by local diffusion corrosion, suggesting that the corrosion area on the aluminum matrix spread insufficiently and showed less corrosion products than at 45 °C. Therefore, the corrosion degree of the copper–aluminum laminated composite plate in the salt-spray corrosion environment aggravated first and then weakened with increasing temperature. However, Wang et al. [23] and Li et al. [24] researched that the corrosion degree ought to exacerbate with the increase of temperature; it was different from the result we achieved in this study, whereby the degree of corrosion hit its maximum at 45 °C and weakened at 50 °C in the salt-spray corrosion environment, and the specific reason is discussed later.

### 3.2. Corrosion Rate Analysis

**Figure 2:**
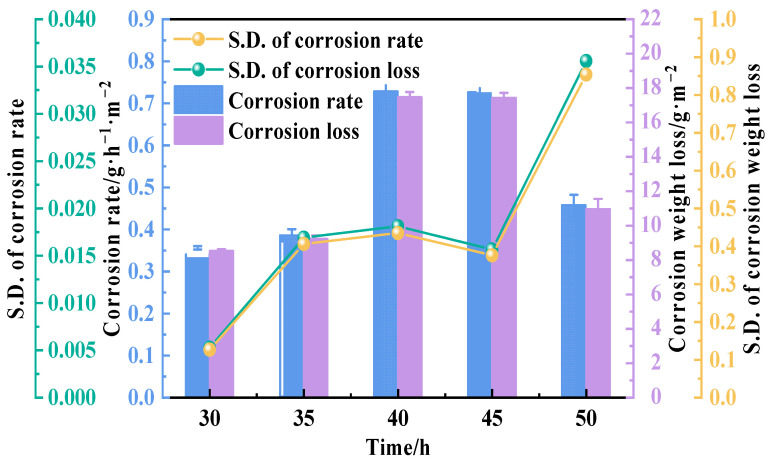
Corrosion rate and corrosion weight loss of copper–aluminum laminated composite plate after salt-spray corrosion at different temperatures for 24 h.

In order to further study the influence of temperature on the corrosion degree in a neutral salt-spray environment, the corrosion weight loss and corrosion rate were measured and calculated at 30, 35, 40, 45, and 50 °C, respectively. Figure 2 shows that the corrosion rate first accelerated from 0.355 g/m^2^·h to a peak value of 0.728 g/m^2^·h with the increasing temperature from 30 to 45 °C and then decreased to 0.457 g/m^2^·h when the temperature reached 50 °C. The changing of the corrosion rate was consistent with the observed morphologies shown in Figure 1. The above results demonstrated that the corrosion rate of the copper–aluminum laminated composite plate was greatly affected by temperature. According to the Nernst equation, as shown in Equation (1), the activity of the reducing ion was proportional to the temperature; therefore, it was readily understood that the corrosion rate would increase when the Cl^−^ activity was promoted by a higher temperature [25]. However, the corrosion rate decreased instead of increasing at 50 °C, thus illustrating that the corrosion rate was not determined by corrosion ion activity exclusively.

(1) $$E=E^{\theta }-\frac{RT}{nF}\ln\frac{a_{\mathrm{Red}}}{a_{\mathrm{Ox}}}$$

where *E* is the electrode potential, and the unit is volts; *E*^θ^ is the standard electrode potential relative to H, and the unit is volts; *R* is the gas constant, which is 8.3143 J/K^−1^·mol^−1^; *T* is the absolute temperature, and the unit is Kelvin; *n* is the number of electrons transferred; and *F* is the Faraday constant, usually 96,500 Coulombs.

### 3.3. XPS Analysis of Corrosion Products

**Figure 3:**
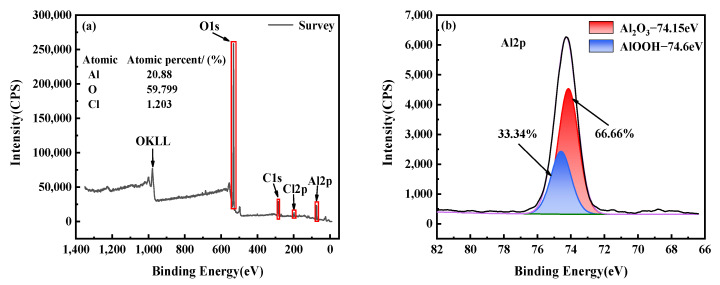
XPS spectrum of corrosion products of salt-spray corrosion at 35 °C: (**a**) XPS total spectrum and (**b**) Al2p spectra of corrosion products.

XPS was utilized to identify the elements of the corrosion products formed on the surface of the copper–aluminum laminated composite plate after salt-spray corrosion at 35 °C, as shown in Figure 3. The total element spectrum observed in Figure 3a indicated that O and Al were present. Therefore, the main product of the salt-spray corrosion process was the oxide of aluminum, which suggested that the metal copper as the cathode hardly participated in the reaction. From the average relative contents of the three elements, O, Al, and Cl, that were detected from the surface corrosion products, we could see that the content ratio of Al and O was basically about 1:3. The above result corresponds to the peak in Figure 3b that represents Al(OH)_3_ (74.3 eV).

To deeply understand the components of the corrosion products, the detected element, Al, of the copper–aluminum laminated composite plate was subjected to peak separation treatment by using CasaXPS software. The characteristic orbital of Al2p is shown in Figure 3b; it was composed mainly of the 66.66% Al_2_O_3_ (74.15 eV) and 33.34% AlOOH (74.6 eV). Compared with metal aluminum (72.6 eV), the binding energy of the Al2p peak in the oxide increased, proving that the binding energy between metal aluminum atoms was weakened, resulting in easier formation of aluminum oxide. The possible electrochemical reactions are discussed later.

### 3.4. Formation and Process of Corrosion Products

The reaction of the copper–aluminum composite plate in the salt-spray corrosion environment was also an electrochemical reaction, so the general equation of the corrosion reaction of the copper–aluminum composite plate was as follows:

(2) $$\mathrm{Al}+2H_{2}O+O_{2}\to {\mathrm{Al}}^{3+}+4{\mathrm{OH}}^{-}+4e^{-}$$

Equation (2) can be divided into two steps:

(3) $$\mathrm{Anode}:\;\mathrm{Al}\to {\mathrm{Al}}^{3+}+{3e}^{-}$$

(4) $$\mathrm{Cathode}:\;2H_{2}O+O_{2}+4e^{-}\to 4{\mathrm{OH}}^{-}$$

**Figure 4:**
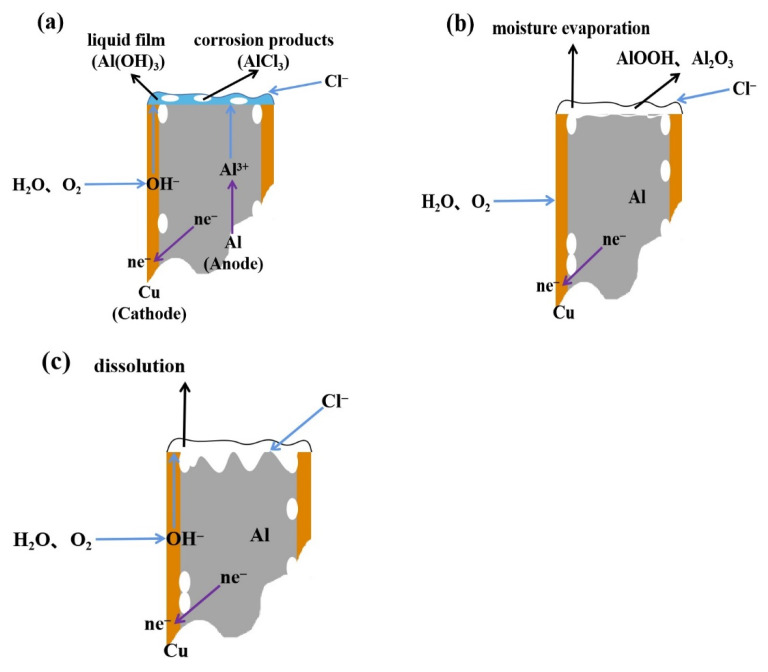
Schematic diagrams for electrochemical corrosion process of copper–aluminum laminated composite plate. (**a**) The process of generating corrosion product, (**b**) The process of forming corrosion product film and (**c**) The process of dissolving corrosion product and corrosion product film.

The electrochemical corrosion process of the copper–aluminum laminated composite plate is shown in Figure 4. According to Equation (5), a dense oxide film would be formed on the surface of the metal aluminum before the corrosion started when the copper–aluminum laminated composite plate was exposed to the atmosphere from Figure 4a. In the early stage of corrosion, since the radius of Cl^−^ ions was smaller than O^2−^, Cl^−^ replaced the oxygen ions in aluminum oxide. Oxygen and active anions competed for adsorption points on the metal surface, destroying the local passivation film on the surface of the material. This was consistent with the pitting nucleation theory studied by Stefan et al. [26]. Meanwhile, AlCl_3_ could react with the exposed local aluminum matrix by Equation (6), causing the copper–aluminum laminated composite plate to corrode. The generated corrosion products and dissolved metal cations were reacted with the cathode to form a layer of metastable liquid film on the surface of aluminum, as shown in Equation (7), which was equivalent to hydration. In addition, part of the metastable liquid film was decomposed into ions, and the remaining part covered the surface of aluminum. It is shown in Figure 4b that temperature would cause water to evaporate, resulting in the formation of AlOOH and Al_2_O_3_ as corrosion products, as shown in Equations (8) and (9). Moreover, aluminum oxide reacted with a small amount of water in Equation (10), causing the content of AlOOH to increase, as was consistent with the XPS results (Figure 3). Corrosion products were consumed by Cl^−^ again, forming porous AlCl_3_, which was easily soluble in water from Figure 4c. When covering the surface of the aluminum matrix, it was easy to fall off, causing the aluminum matrix to be exposed and be corroded again. This was repeated until the aluminum matrix was completely consumed.

(5) $$4\mathrm{Al}+{3O}_{2}\to {2\mathrm{Al}}_{2}O_{3}$$

(6) $$\mathrm{Al}+{3\mathrm{Cl}}^{-}\to {\mathrm{AlCl}}_{3}$$

(7) $${\mathrm{AlCl}}_{3}+3{\mathrm{OH}}^{-}\to \mathrm{Al}{\left(\mathrm{OH}\right)}_{3}+3{\mathrm{Cl}}^{-}$$

(8) $$2\mathrm{Al}(\mathrm{OH}{)}_{3}\to {\mathrm{Al}}_{2}O_{3}+3H_{2}O$$

(9) $$\mathrm{Al}(\mathrm{OH}{)}_{3}\to \mathrm{AlOOH}+H_{2}O$$

(10) $${\mathrm{Al}}_{2}O_{3}+H_{2}O\to 2\mathrm{AlOOH}$$

### 3.5. AC Impedance Spectrum Analysis

**Figure 5:**
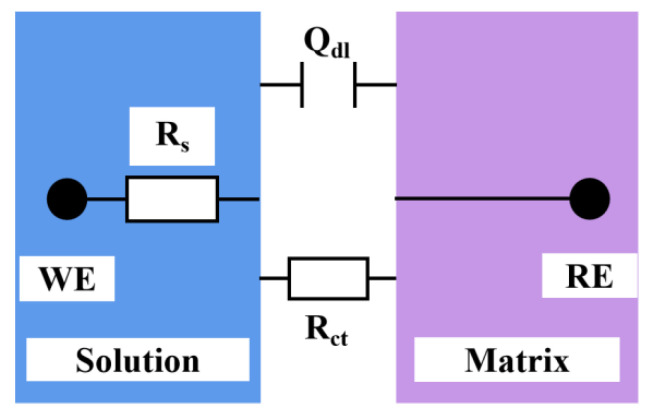
Electrochemical impedance equivalent circuit diagram.

To understand the effect of temperature on the corrosion resistance of the copper–aluminum laminated composite plate, an AC impedance measurement was carried out to investigate the corrosion performance after attaining a stable OCP, and the Nyquist curve and Bode curve were obtained by fitting with ZSimpWin software (3.60, 2014, Ametek Scientific Instruments, Berwyn, PA, USA). The AC impedance spectrum parameters were simulated by using the equivalent electrical circuit models (Figure 5) composed of *R*_p_, *R*_s_, and *Q*_dl_ circuit, which provided the best fitting results. Due to the fact that pure capacitance hardly applies to real electrochemical processes [27], the non-ideal capacitance was replaced by a constant phase angle element, *Q* [28], which represents an electric double-layer capacitor. *R*_s_ meant solution resistance, and *R*_p_ stood for polarization resistance or charge-transfer resistance. *R*_p_ was a quantitative parameter which explained the ease of Cl^−^ transferring in the solution, and it could be an evaluation criterion for the corrosion resistance of materials [29].

The relevant AC impedance spectrum parameters are revealed in Table 1; the fitted equivalent circuit R(QR) indicated that the corrosion process only exhibited capacitive arc-resistance characteristics, and it was mainly controlled by activation which was a process through which charges or other substances were transported through the corrosion product film. The surface of the metal aluminum was negatively charged and positively charged on the solution layer on the metal surface; both of them constituted an electric double-layer capacitor, *Q*. Therefore, the impedance spectrum measured on the surface of the electrode showed a good dispersion effect. The value of the dispersion coefficient, n, depended on the roughness of the electrode surface and the unevenness of the corrosion current distribution. Due to the uneven electric field distribution on the electrode surface, the electrode reaction would produce a relaxation process. Table 1 suggested that, when the temperature was risen, *R*_p_ gradually decreased. In other words, the increase in temperature led to a reduction in the resistance that hindered the movement of ions that aggravated the material corrosion, signifying that the corrosion resistance of the material was reduced. Therefore, the temperature would affect the mobility of Cl^−^ in the solution, which, in turn, affected the corrosion resistance of the composite material.

**Figure 6:**
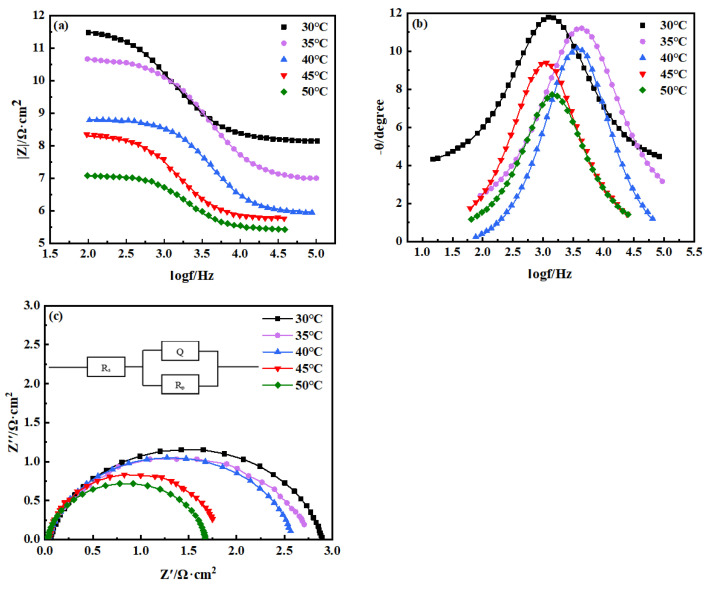
Electrochemical impedance spectroscopy of copper–aluminum laminated composite plate at different temperatures: (**a**) module diagram, (**b**) phase diagram, and (**c**) Nyquist curve.

Figure 6 shows the electrochemical impedance spectroscopy of the copper–aluminum laminated composite plate at different temperatures. Figure 6a is the model diagram in the bode; the impedance value in the low-frequency zone was precisely related to the solution resistance [30]. Figure 6b displays the relationship between phase angle and frequency. The impedance and phase angle declined with the temperature increase, indicating that the corrosion-resistant performance gradually weakened. Table 2 expresses the difference between the low-frequency impedance and the simulated *R*_s_, which could be an index to evaluate the ion mobility in solution, also called transfer resistance. Both the difference and *R*_s_ decreased slightly with the increase of temperature; such a decline indicated the degradation in corrosion resistance. Figure 6c exhibits a capacitive arc semicircle for which the radius is related to the double-layer capacitance and charge-transfer resistance. As the temperature increased, the radius diminished, indicating that the corrosion resistance of the material degenerated. The Nyquist curve corresponded to an evident time constant in the bode diagram, which represented that the corrosion products formed in the electrochemical corrosion process of the copper–aluminum laminated composite plate were very loose and cannot protect the aluminum surface. Therefore, the consequence of the impedance spectrum implied that corrosion rates of copper–aluminum laminated composite plate increased with the temperature in the electrochemical corrosion process.

### 3.6. Polarization Curve Analysis

**Figure 7:**
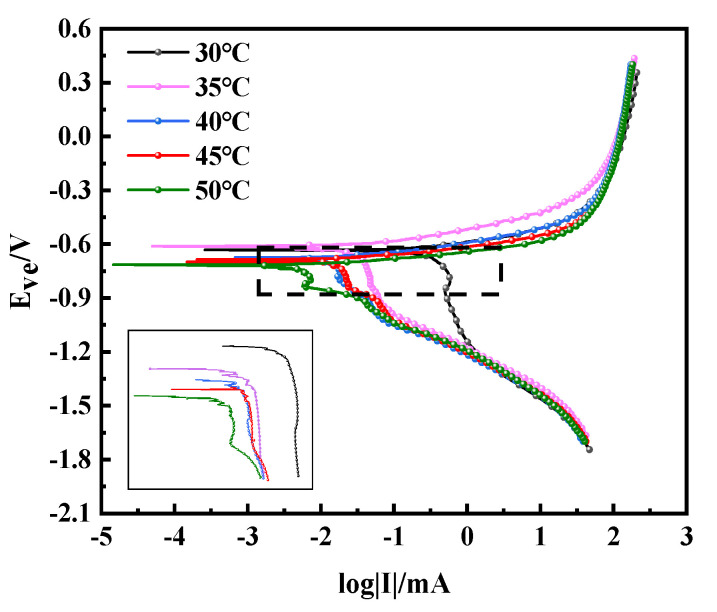
Tafel curve of copper–aluminum laminated composite plate at different temperatures.

Figure 7 shows the Tafel curve of the copper–aluminum laminated composite plate at various temperatures. It could be seen that the anode did not undergo self-passivation, meaning that the Tafel behaved as activation control. With the rising of temperature, the corrosion potential negatively shifted, suggesting that the rising of temperature promotes the total reaction process. Moreover, it indicated a pseudo-passive behavior on the cathode, due to the fact that the corrosion product film covered the surface of aluminum. All of the corrosion kinetic parameters obtained by linear fitting or the Tafel extrapolation are presented in Table 3. Since the corrosion potential as a thermodynamic parameter only played a role in predicting the corrosion rate of the sample, the corrosion current density was usually used to judge the corrosion rate. We can see from Table 3 that the corrosion potential (*E*_corr_) decreased and the corrosion current density (*J*_corr_) increased with increasing temperature, leading to the elevation of the corrosion rate and the reduction of the corrosion resistance. The anode polarizability was smaller than the cathode polarizability, as shown in Table 3; however, both of them were increased with the increase of temperature. It was clear from Figure 7 that the cathode changed more obviously, so it emphasized that the corrosion was mainly controlled by the oxygen absorption reaction of the cathode; it was controlled by the content of oxygen and water. In consequence, it could be judged from the Tafel curve and the fitting value that the corrosion resistance of the copper–aluminum laminated composite plate decreased as the temperature increased.

## 4. Discussion

**Figure 8:**
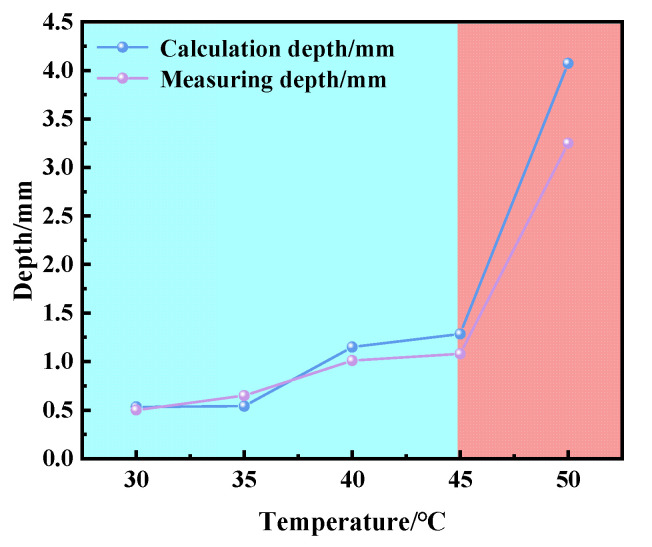
Corrosion-pit-depth-and-temperature relationship diagram.

**Figure 9:**
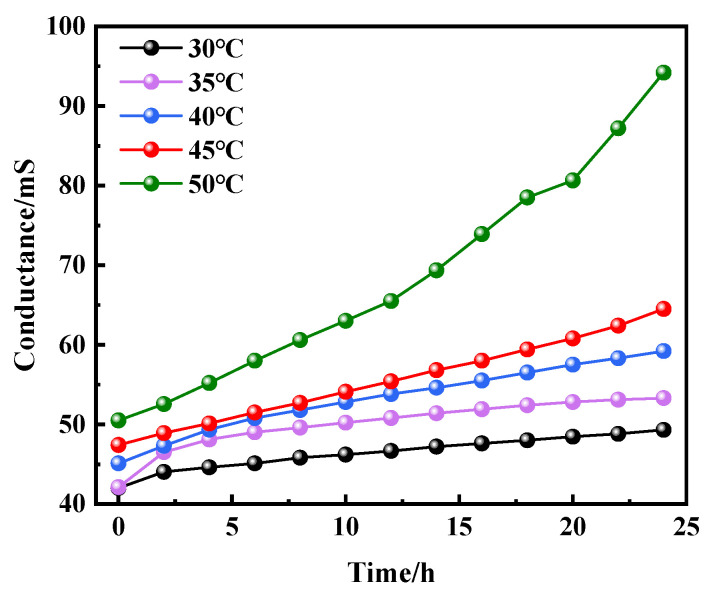
Conductivity vs. time curve of 3.5% NaCl solution at different temperatures.

The corrosion rate of the copper–aluminum laminated composite plate increased in salt-spray environment or electrochemical environment, while the corrosion resistance decreased, when the temperature was increased from 30 to 45 °C. According to the Nernst equation (Equation (1)), the Cl^−^ activity increased as the temperature increased. Therefore, the low Cl^−^ activity at 30 °C in the salt environment could only replace limited O^2−^, causing few pitting pits on the aluminum matrix near the interface, as shown in Figure 1a. This limited surface damage indicated that the oxide film was relatively intact, which provided a preferable anti-corrosion protection ability and resulted in the corrosion rate being reduced for the surface of the aluminum matrix. The activity of Cl^−^ was enhanced by the increasing temperature, which caused the replacement of oxygen atoms in the oxide film on the surface of the aluminum matrix to be accelerated continuously; subsequently, the pitting pits were further expanded, as shown in Figure 1b. With the constant diffusion of Cl^−^, crevice corrosion gradually formed along the interface. When the temperature was increased to 40–45 °C, the passivation film was completely destroyed by Cl^−^. Cl^−^ reacted with the exposed anode aluminum matrix to generate corrosion products (AlCl_3_) quickly. AlCl_3_ reacted with the OH^−^, which was generated at the cathode to form a liquid film Al(OH)_3_ first, and then converted to AlOOH and Al_2_O_3_. Due to the fact that electrochemical corrosion was conducted in the solution, it is difficult for the corrosion products to adhere to the surface of the aluminum matrix for protection, wherefore obvious depressions are formed on the surface of the material. By combining Formulas (11)–(13) [31], we can calculate the corrosion depth, as shown in Formula (14). The calculated and measured corrosion depth showed a consistent trend, although the values were slightly deviated. This deviation could be contributed to the unsmooth surface of the pits or the non-ideal experimental environment, such as the roughness of the sample. Figure 8 shows the curve of electrochemical-corrosion-pit depth versus temperature; it can be seen that the depth of the corrosion pit increased substantially at 50 °C, from 0.5 mm to more than 3 mm. The degree of corrosion deepened with increasing temperature, the same law bound as the solution ion mobility in Figure 9. It was observed that the ion mobility was greatly increased, especially at 50 °C. The conductivity of the solution was proportional to the temperature, as shown in Formula (15), signifying that the corrosion rate of the copper–aluminum laminated composite plate was rapidly increasing as the temperature rose. However, in the salt-spray environment, the corrosion rate decreased at 50 °C.

(11) $$I_{K}=\frac{\beta_{a}\times \beta_{c}\times \Delta I}{2.3\left(\beta_{a}+\beta_{c}\right)\times \Delta E}=\frac{B}{R_{p}}$$

(12) $$I_{K}=\frac{nF}{W}v$$

(13) $$D=\frac{8.76\times v}{\rho }$$

(14) $$D=\frac{8.76\times \beta_{a}\times \beta_{c}\times W}{2.3\left(\beta_{a}+\beta_{c}\right)\times n\times F\times R_{p}\times \rho }$$

where *I*_k_ is the corrosion current; *R*_p_ is the polarization resistance, and the unit is Ω·cm^2^; *β*_a_ and *β*_c_ are the Tafel constants of the anode and cathode, respectively; *W* is the atomic mass; *v* is the corrosion rate; and *ρ* is the material density.

(15) A=e×(c×n×G)

where *c* is the ion concentration, *n* is the charge number, and *G* is the ion electromobility.

In the salt-spray environment, the water as a carrier for Cl^−^ on the surface of the material significantly evaporated when the temperature reached 50 °C, which led to the diffusion of Cl^−^ being reduced. The products of AlCl_3_ and Al(OH)_3_ were tightly attached on the surface of the aluminum, due to dehydration, as shown in Equations (8) and (9). AlOOH showed a fibrous morphology which could hinder the corrosion caused by Cl^−^ and provide strong protection to the matrix [32,33]. Therefore, the corrosion process was slowed down, and the corrosion rate was reduced when the temperature reached 50 °C. Qiao [34] reported a similar conclusion that high temperature accelerated the evaporation process of the NaCl electrolyte layer, which, covered on the surface of the material, made the electrolyte membrane thinner subsequently. However, the corrosion resistance tested by electrochemical technology suggested that it was monotone decreasing when the temperature increased. In contrast to the salt-spray corrosion, the electrochemical corrosion proceeded in solution, which can provide sufficient water and oxygen to the surface of the materials to ensure that the corrosion is uninterrupted, even when the temperature reached 50 °C. Therefore, the corrosion behavior of copper–aluminum laminated composite plates in different environments was inseparable from water and oxygen. The corrosion mechanism was same for both the electrochemical process and salt-spray corrosion process when the temperature was 30–45 °C. However, when the temperature was increased to 50 °C, the corrosion rate increased in electrochemical process, but decreased in salt-spray corrosion.

## 5. Conclusions

Temperature has different effects on the corrosion behavior and corrosion resistance of copper–aluminum laminates in electrochemical corrosion and salt-spray corrosion environments, which mainly depends on whether there is enough moisture and oxygen in the environment. In the salt-spray corrosion environment, when the temperature becomes high, the evaporation of water leads to the reduction of the medium carrier and the insufficient amount of dissolved oxygen; therefore, when the copper–aluminum laminate composite plate is in a relatively dry or oxygen-deficient environment, the corrosion resistance is first decreased and then increased. The corrosion resistance reached the lowest at 45 °C, and then increased at 50 °C, while the corrosion rate was the highest at 45 °C, which was 0.728 g/m^2^·h; after that, it decreased when the temperature rose to 50 °C. However, in the electrochemical corrosion environment, due to the sufficient oxygen content and the humid environment, the corrosion resistance of the copper–aluminum laminated composite plate was reduced as the temperature rose.

## Figures and Tables

**Table 1:** Values of circuit components in the equivalent circuit of the AC impedance spectrum of the copper–aluminum laminated composite plate.

Temperature/°C	*R*_s_/Ω·cm^2^	*Q*_dl_/Ω^−1^·cm^−2^·s^n^	*Q*-*n*	*R*_p_/Ω·cm^2^
30	7.127	5.046 × 10^−5^	0.8228	4.679
35	6.910	5.665 × 10^−5^	0.8465	3.795
40	6.100	7.269 × 10^−5^	0.9136	2.918
45	6.005	8.407 × 10^−5^	0.9277	2.584
50	5.411	6.501 × 10^−5^	0.9699	1.657

**Table 2:** Difference between the low-frequency impedance and the simulated solution resistance, *R*_s_.

Temperature/°C	100 Hz Impedance/Ω·cm^2^	*R*_s_/Ω·cm^2^	Difference/Ω·cm^2^
30	11.5	7.127	4.373
35	10.7	6.910	3.79
40	8.7	6.100	2.6
45	8.4	6.005	2.395
50	7.07	5.411	1.659

**Table 3:** Fitting values of Tafel curve of copper–aluminum laminated composite plate at different temperatures.

Temperature/°C	*E*_corr_/mV	*J*_corr_/μA·cm^−2^	*β*_a_/mV	*β*_c_/mV
30	−643.664	13.007	63.5	287.5
35	−648.349	14.664	49.8	301.2
40	−746.500	16.562	83.3	398.5
45	−765.462	21.134	82.7	421.9
50	−1093.796	27.211	197.8	485.8

## Data Availability

The raw/processed data required to reproduce these findings cannot be shared at this time, as the data also form part of an ongoing study that cannot be shared at this time, due to technical or time limitations.
